# Association between ozone and influenza transmissibility in China

**DOI:** 10.1186/s12879-023-08769-w

**Published:** 2023-11-06

**Authors:** Jiao Yang, Ting Zhang, Liuyang Yang, Xuan Han, Xingxing Zhang, Qing Wang, Luzhao Feng, Weizhong Yang

**Affiliations:** 1 School of Population Medicine and Public Health, Chinese Academy of Medical Sciences & Peking Union Medical College, Beijing, China; 2 State Key Laboratory of Respiratory Health and Multimorbidity, Beijing, China; 3 Key Laboratory of Pathogen Infection Prevention and Control (Peking Union Medical College), Ministry of Education, Beijing, China; 4 Department of Management Science and Information System, Faculty of Management and Economics, Kunming University of Science and Technology, Kunming, Yunnan China

**Keywords:** Influenza, Transmissibility, Flu, Ozone

## Abstract

**Background:**

Common air pollutants such as ozone (O_3_), sulfur dioxide (SO_2_), nitrogen dioxide (NO_2_), and particulate matter play significant roles as influential factors in influenza-like illness (ILI). However, evidence regarding the impact of O_3_ on influenza transmissibility in multi-subtropical regions is limited, and our understanding of the effects of O_3_ on influenza transmissibility in temperate regions remain unknown.

**Methods:**

We studied the transmissibility of influenza in eight provinces across both temperate and subtropical regions in China based on 2013 to 2018 provincial-level surveillance data on influenza-like illness (ILI) incidence and viral activity. We estimated influenza transmissibility by using the instantaneous reproduction number (${R}_{t}$) and examined the relationships between transmissibility and daily O_3_ concentrations, air temperature, humidity, and school holidays. We developed a multivariable regression model for ${R}_{t}$ to quantify the contribution of O_3_ to variations in transmissibility.

**Results:**

Our findings revealed a significant association between O_3_ and influenza transmissibility. In Beijing, Tianjin, Shanghai and Jiangsu, the association exhibited a U-shaped trend. In Liaoning, Gansu, Hunan, and Guangdong, the association was L-shaped. When aggregating data across all eight provinces, a U-shaped association was emerged. O_3_ was able to accounted for up to 13% of the variance in ${R}_{t}$. O_3_ plus other environmental drivers including mean daily temperature, relative humidity, absolute humidity, and school holidays explained up to 20% of the variance in ${R}_{t}$.

**Conclusions:**

O_3_ was a significant driver of influenza transmissibility, and the association between O_3_ and influenza transmissibility tended to display a U-shaped pattern.

**Supplementary Information:**

The online version contains supplementary material available at 10.1186/s12879-023-08769-w.

## Introduction

In different climates, influenza shows a variable epidemic pattern. For example, in temperate climates, seasonal epidemics mainly occur during winter [1]; in subtropical climates, influenza often shows two annual peaks, in winter and summer; and in tropical climates, influenza outbreaks may occur irregularly throughout the year [1, 2]. The difference in influenza epidemic patterns may directly or indirectly affect the response strategies, such as vaccination and the allocation of medical resources [3].

Many factors affect the spread of influenza including human mobility [4], humidity [5, 6], non-pharmaceutical interventions [7], air pollution [8], the types of virus, and the immunity of the population [9]. Of these, ambient pollutants have received an increasing amount of attention. One study in Australia showed that high concentrations of ozone (O_3_) and PM_10_ were significant risk factors for pediatric influenza [8]. In Beijing, China, ambient PM_2.5_ concentrations were significantly associated with influenza-like illness (ILI) incidence risk during the flu season across multiple age groups [10]. Compared with the occurrence of influenza, evidence of the relationship between ambient pollution and influenza transmissibility remains limited. The transmissibility index of influenza can be characterized by the reproduction number (${R}_{t}$), defined as the average number of secondary infections caused by a typical single infectious individual at time t; a higher ${R}_{t}$ value indicates higher transmissibility. Ali et al. reported that O_3_ is a significant driver for influenza transmissibility and has an L-shaped relationship with ${R}_{t}$ in Hong Kong based on data for all types/subtypes [11]. However, as Hong Kong is located in the subtropic, differences in climate may affect such relationships in other regions. Accordingly, exploring the relationship between O_3_ and ${R}_{t}$ in different climates is urgently needed to clarify the potential impact of O_3_ on influenza transmissibility.

In China, most of the northern provinces have a temperate climate, while most of the southern provinces are subtropical. Therefore, in this study, we selected four provinces each in northern and southern China to examine the impact of O_3_ on influenza transmission.

## Methods

### Data source: 5 years of data from 2013 to 2018

Hourly ambient temperature and dew point temperature data for each province were obtained from the National Centers for Environmental Information (Global Surface Summary of the Day—GSOD. https://www.ncei.noaa.gov/access/search/data-search/global-hourly?bbox=40.563,115.742,39.252,117.052&pageNum=1, Accessed 4 August 11 2021). Using the R package “humidity” (R software, version 4.2.1), we calculated hourly relative and absolute humidity. The daily values for temperature, relative humidity (RH) and absolute humidity (AH) were determined by taking the arithmetic mean of their respective hourly values for each day.

Daily concentrations of O_3_ in the eight provinces were obtained from the China High Air Pollutants (CHAP) dataset [12]. CHAP is a long-term, full-coverage, high-resolution, and high-quality datasets of ground-level air pollutants for China. This dataset produced high-quality daily O_3_ concentrations on 10 km × 10 km grid scale, derived from big data (e.g., ground-based measurements, satellite remote sensing products, atmospheric reanalysis, and model simulations), by using artificial intelligence. Its cross-validation coefficient of determination, a root-mean-square error (RMSE), and a mean absolute error (MAE) for daily O_3_ concentrations were found to be 0.87, 17.10 $ug/{m}^{3}$ and 11.29 $ug/{m}^{3}$ respectively when compared with data from ground stations [13, 14]. For provincial-level, the daily O_3_ concentration was calculated by taking the arithmetic mean of values from each 10 km x 10 km grid. Information about holiday-related school closures, including public holidays, summer holidays, Chinese New Year holidays and winter holidays, was also collected. Weekly ILI and viral-detection rate data for each province were obtained from the Chinese National Influenza Surveillance Network. Based on previous studies [15–20], proxy measures for the weekly incidence rate were obtained by multiplying the ILI percentage among patients visiting sentinel hospitals with the proportions of influenza-positive specimens, which is referred to as influenza rate. This proxy is considered a precise representation of the activity of influenza infection [21, 22]. We multiplied the weekly incidence rate by a constant (10,000) representing the inverse of the coverage of the sentinel sites in the studied provinces, and rounded the resulting values to the nearest integers to obtain a time series of weekly incidence rate counts (ILI + counts) [23]. Due to differences in the epidemiological characteristics of influenza in southern and northern China [5, 24], we conducted analysis by region and constricted in 8 provinces and municipalities (Fig. S1). We selected these locations based on the availability of influenza surveillance data and O_3_ concentration during the study period. Beijing, Tianjin, Shanghai and Jiangsu have relatively high O_3_ concentrations, while Hunan, Guangdong, Liaoning and Gansu have relatively low O_3_ concentrations [12].

Influenza epidemics were defined as outbreaks exceeding the epidemic threshold for at least seven consecutive weeks or more. The epidemic threshold was determined as the 50th percentile of all the non-zero weekly incidence rate counts over the study period [23]. Cubic spline interpolation was employed to convert the weekly influenza rate and ILI + counts into daily influenza rate and ILI + counts, which were subsequently used to estimate transmissibility [22, 23]. Cubic spline interpolation operates by constructing piecewise cubic polynomial functions that smoothly connect each weekly data point. By doing so, it generates interpolated values for daily data points. These functions ensure not only that the curve passes through each weekly data point, but also that the transitions between segments are continuous and smooth.

### ${{\varvec{R}}}_{{\varvec{t}}}$ and adjusted ${{\varvec{R}}}_{{\varvec{t}}}$ estimation

Using the Bayesian framework applied to the branching process model, ${R}_{t}$ was estimated as proposed by Cori et al. [25]. ${R}_{t}$ serves as a measure of transmissibility. A gamma distribution, characterized by a mean of 2.6 days and a standard deviation of 1.5 days, was assumed for the serial interval distribution [26]. As the epidemic progresses, there is an observable decline in the number of susceptible individuals in the population, resulting in a gradual decrease of ${R}_{t}$. To accommodate this change, the adjusted ${R}_{t}$ was calculated using the methodology outlined by Ali et al. [23]. Further details on the ${R}_{t}$ estimation process can be found in the Supplementary Material.

### Exploratory data analysis using *R*_*t*_

To accommodate reporting lags ranging from 0 to 14 days, we evaluated the best functional relationship between ${R}_{t}$ and every potential driver in each province, utilizing both exponential and power univariate regression models [22, 23]. The selection of significant drivers with best-fitted functions was based on variations in the Akaike information criterion (ΔAIC):

$$\Delta_i={\Delta\mathrm{AI}C}_{\mathrm i}={\mathrm{AIC}}_{\mathrm i}-{\mathrm{AIC}}_{\mathrm{main}}$$

where $i=$ exponential or power form of the association and:

$${\mathrm{AIC}}_{min}={\mathrm{AIC}}_{exponential}{,\mathrm{AIC}}_{power}$$

In addition, we employed aggregated data from the eight provinces to construct a general model that investigates the correlation between ${R}_{t}$ and its various drivers. Subsequently, we executed a permutation analysis on 1,000 dummy or null scenarios using regression models to ascertain if the relationship between ${R}_{t}$ and O_3_ was due to chance. The results of this investigation were compared with the true time-series dataset.

### Quantifying the impacts of drivers on ${{\varvec{R}}}_{{\varvec{t}}}$

We constructed three multivariable regression models to explore the impacts of the different drivers on ${R}_{t}$. “Model 1” evaluated the impacts of the depletion in susceptibility over time and/or inter-epidemic effects on ${R}_{t}$; “Model 2” incorporated the additional effect of O_3_; and “Model 3” further took into account school holidays, temperature, relative humidity, and absolute humidity. Using the best lagged model and distributed lag non-linear models (DLNMs), we calculated R^2^ to quantify the extent to which these influencing factors explained the variation in ${R}_{t}$. The formula of the DLNMs model is

1 $$log\left(E\left[{y}_{t}\right]\right) = \alpha + {\beta }_{1}{T}_{t,l} \left({temp}_{t}\right) + {\beta }_{2}{T}_{t,l} \left({AH}_{t}\right) + {\beta }_{3}{T}_{t,l} \left({RH}_{t}\right)+ {\beta }_{4}{T}_{t,l} \left({O3}_{t}\right) +ns \left(cu{m}_{{inci}_{t}},df =3\right) +ns\left({holidays}_{t},df\right) =3$$

${\mathrm{wherey}}_{\mathrm\,t}$ is the expected ${R}_{t}$ on day t. ${\beta }_{i}$ is the regression coefficient value for each factor on${R}_{t}$. ${T}_{t,l}$ is the cross-basis function of the each factor (temperature,relative humidity, absolute humidity and O_3_) level at day t and lag l, and the basis function is “poly”, and the natural spline function with a degree of freedom of 3 is used for the lag dimension; ns is the natural spline basis function; df is the degree of freedom; In addition, we controlled the effects of depletion of susceptibles ($cum\_inci$) by using a natural cubic spline with 3 df.

The formula of the best lagged model is

2 $${\mathrm y}_{\mathrm t}=\mathrm e^{{\mathrm\beta}_0}+{\mathrm\beta}_1{\mathrm x}_1+{\mathrm\beta}_2\mathrm x_2^2+{\mathrm\beta}_3{\mathrm x}_2+{\mathrm\beta}_4\mathrm x_2^2\cdots$$

where ${y}_{t}$ is the expected ${R}_{t}$ on day t. ${\beta }_{0}$ is the intercept term. ${\beta }_{1}$ is the coefficient for the factor ${x}_{1}$. ${\beta }_{2}$ is the coefficient for the squared term of factor ${x}_{1}$. Similarly, each factor has two associated coefficients: one for the factor itself and another for its squared term.

The best lag model (i.e., those with a specific lag and the largest *R*^*2*^ value) and the distributed lag non-linear models (DLNMs, the R package “dlnm”, version 2.4.7) were utilized to compute the *R*^*2*^ values. The difference between the *R*^*2*^ values of Model 1 and Model 2 quantified the effect of O_3_ on ${R}_{t}$. The *R*^*2*^ values of Model 3 gauged the impact of all factors on ${R}_{t}$. The DLNMs accounts for the overall effect of the multi-day distribution, rather than presenting results solely for the most optimal lag. This distributed modeling approach also factors in the probability of infection from previous days (equivalent to at least the average generation time) to assess the transmissibility, ${R}_{t}$.

## Results

### Background characteristics by province

**Fig. 1:**
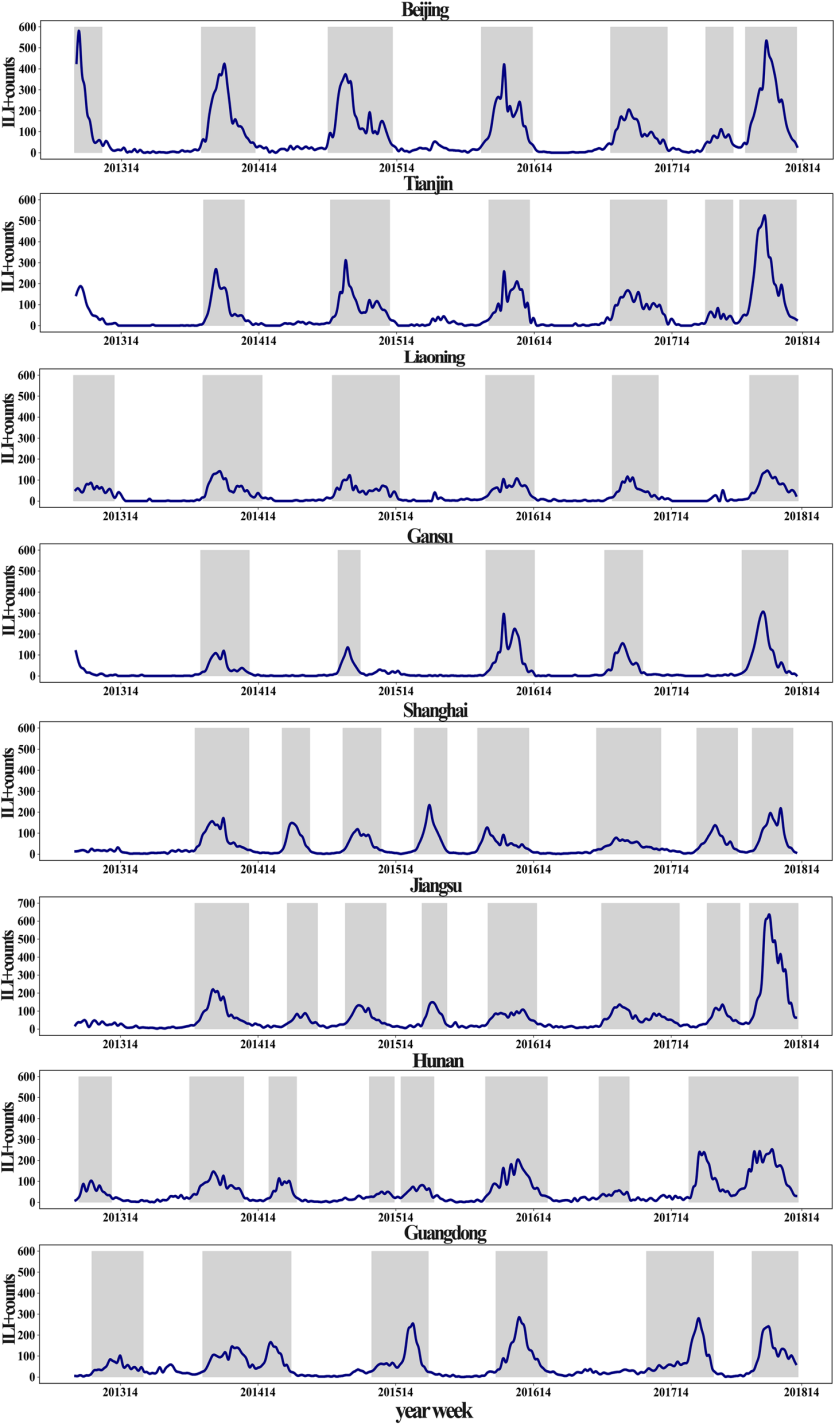
Weekly influenza activity as ILI + counts (blue lines) along with the predefined epidemics (gray shaded area) in eight different provinces in China from 2013 to 2018

As shown in Fig. 1, during the study period (2013–2018), a total of 54 distinct influenza epidemics were identified (seven for Beijing, six for Tianjin, six for Liaoning, five for Gansu, eight for Shanghai, eight for Jiangsu, eight for Guangdong, and six for Hunan) with different lengths and patterns (i.e., single or double peaks). Table 1 presented the summary statistics of influenza rate, ILI + counts, ${R}_{t}$, O_3_, daily temperature, and humidity in the eight provinces. The areas with daily ILI + counts ranked from high to low were Jiangsu, Guangdong, Beijing, Hunan, Shanghai, Tianjin, Liaoning and Gansu. The areas with daily median ozone concentrations, ranked from high to low were Shanghai, Gansu, Jiangsu, Liaoning, Beijing, Tianjin, Guangdong, Hunan. However, for the 75th percentile of daily O_3_ concentrations, the ranking from high to low is Shanghai, Beijing, Tianjin, Jiangsu, Liaoning, Gansu, Guangdong, and Hunan. The median values of ${R}_{t}$ for all the epidemics was 1.0, with the minimum values ranging from 0.7 to 0.8 and the maximum values from 1.23 to 2.0. The climate is colder and drier in the Northern provinces.

**Table 1:** Descriptive Statistics (including median, min–max for ${R}_{t}$ and median and IQR for influenza rate, ILI + counts, O_3_, daily temperature, and humidity) across eight provinces in northern and southern China from 2013 to 2018

Provinces		${R}_{t}$	Influenza rate (‰)^a^	ILI + counts^b^	O_3_ ($ug/{m}^{3}$)	Temperature (℃)	Relative humidity (%)	Absolute humidity ($g/{m}^{3}$)
Northern China	Beijing	1.0 (0.8,2.0)	0.3 (0.1,1.2)	32.0 (11.0,116.0)	82.9 (54.0,123.4)	13.6 (1.9,23.4)	48.7 (32.1,67.0)	5.1 (2.2,12.2)
	Tianjin	1.0 (0.7,1.6)	0.2 (0.0,0.8)	18.0 (3.0,82.0)	80.0 (49.4,123.0)	14.1 (2.5,24.1)	52.5 (37.2,67.5)	5.6 (2.8,12.7)
	Gansu	1.0 (0.7,1.5)	0.0 (0.0,0.3)	4.0 (1.0,25.0)	95.4 (76.3,109.1)	10.8 (2.2,18.3)	54.2 (39.2,71.3)	5.5 (2.5,9.7)
	Liaoning	1.0 (0.7,1.5)	0.1 (0.0,0.5)	13.0 (2.0,52.0)	83.2 (60.7,109.9)	10.5 (-2.1,20.8)	58.0 (46.8,69.9)	5.0 (2.3,11.7)
Southern China	Shanghai	1.0 (0.7,1.4)	0.2 (0.1,0.6)	20.0 (7.0,57.0)	96.0 (72.8,124.2)	18.1 (9.4,24.5)	69.2 (59.4,79.1)	10.4 (6.1,17.1)
	Jiangsu	1.0 (0.8,1.4)	0.3 (0.2,0.8)	34.0 (16.0,77.0)	91.1 (68.2,118.0)	16.0 (7.0,23.5)	70.7 (58.9,80.4)	8.7 (4.9,15.6)
	Guangdong	1.0 (0.8,1.3)	0.3 (0.2,0.7)	33.0 (15.0,74.0)	80.1 (64.4,100.3)	23.5 (17.5,27.7)	76.2 (68.2,83.2)	16.2 (10.5,21.9)
	Hunan	1.0 (0.8,1.5)	0.3 (0.1,0.7)	29.0 (12.0,70.0)	77.5 (62.3,93.9)	17.4 (9.6,24.1)	75.4 (63.9,84.4)	10.7 (6.4,17.2)

*IQR* Interquartile Range

^a^ Given that the influenza rate has a very small value, it is denoted in parts per thousand

^b^ ILI + counts = Influenza rate × 10000

### Univariate regression model

We first constructed two univariate non-linear regression functions (exponential and power forms) to explore the associations between each driver and ${R}_{t}$ with lagged values of 0–14 d for each province. AIC differences were used to select the best-fitting function for each driver, and the results are shown in Table S1. In all provinces, an exponential fit was better than that of the power form when fitting each factor and transmissibility. On this basis, we determined the significant influencing factors for influenza transmissibility, and incorporated these into further multivariable regression models.

**Fig. 2:**
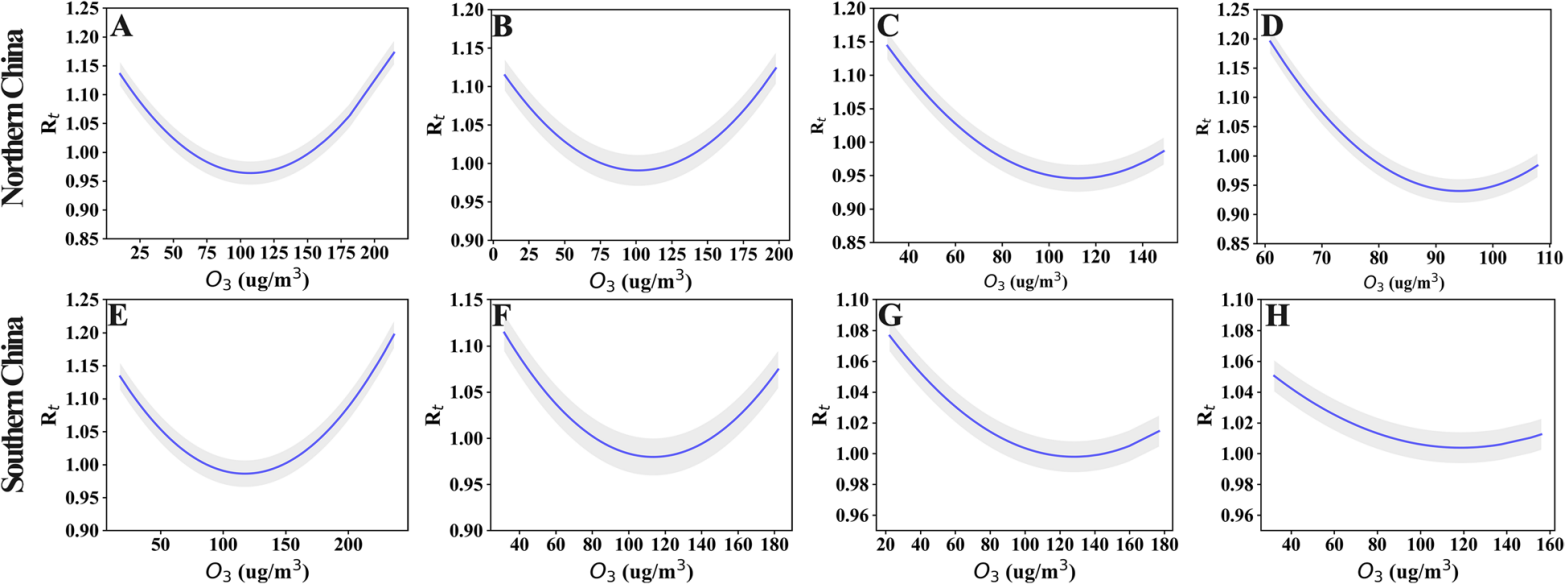
The association between O3 and influenza transmissibility (Rt) of influenza in different provinces (**A**-**H**). **A**-**D** for four provinces in northern China (Beijing, Tianjin, Liaoning and Gansu), (**E**–**H**) for four provinces in southern China (Shanghai, Jiangsu, Guangdong and Hunan). The blue line refers to the R_t, and the gray shading is the 95% confidence interval for the transmissibility

**Fig. 3:**
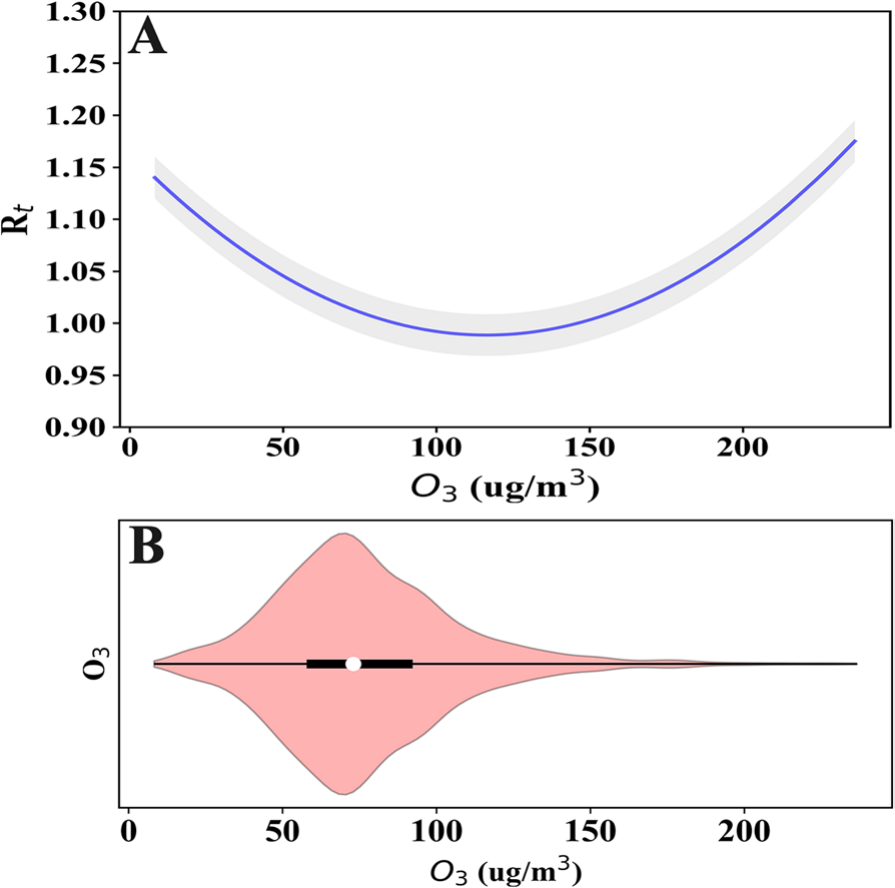
**A** The predicted general U-shaped form (blue line) with 95% CI (shaded region) of association for O3 on influenza transmissibility; (**B**) violin plot of aggregated O3 across all the eight provinces

As shown in Fig. 2, the U-shaped or L-shaped association gave the best-fitting model for the association between O_3_ and influenza transmissibility. In provinces with relatively high O_3_ concentrations (maximum > 180 µg/m^3^), the association between O_3_ and ${R}_{t}$ is more likely U-shaped, such as in Shanghai, Beijing, Tianjin, and Jiangsu. However, in provinces with relatively low O_3_ concentrations (maximum < 180 µg/m^3^), the association between O_3_ and ${R}_{t}$ is more likely to be L-shaped, such as in Liaoning, Gansu, Hunan, and Guangdong. In addition, to further explore whether this U-shaped or L-shaped association was universal, we conducted the same analysis using the aggregated data for eight provinces. The results revealed a persistent U-shaped association (Fig. 3). The permutation test indicated that the true time series of O_3_ explained a significantly larger variance in ${R}_{t}$ compared the null/dummy time series of O_3_ (Table S2). Therefore, O_3_ is a significant driver of influenza transmissibility.

### Quantifying the impacts of different drivers on ${{\varvec{R}}}_{{\varvec{t}}}$

Our multivariate regression model explained 28%–68% of the observed variation in ${R}_{t}$. Notably, a considerable part of the variation was explained by model 1, including the depletion in susceptibility and/or inter-epidemic effects (Table 2). Incorporating O_3_ into model 2 slightly improved the model fit (${R}^{2}$), explaining an additional 1%–13% ($\%\Delta {R}^{2}$) of the variance in ${R}_{t}$ (Table 2) compared with model 1. To control for the depletion in susceptibility, we repeated three multivariate regression analyses with adjusted ${R}_{t}$. The results showed that O_3_ significantly improved the prediction of residual ${R}_{t}$, and further inclusion of other influencing factors only marginally improved the model fit. We used two methods to assess the explanatory power of drivers on ${R}_{t}$, and found that the DLNMs explained a higher proportion of the variation in ${R}_{t}$ than the best lag regression model (Table S3).

**Table 2:** Percentage of the variance of the instantaneous reproduction number (${R}_{t}$) explained by the drivers, across respective provinces from 2013 to 2018. The results are based on the distributed lag model (DLNMs) with lags of 0–2 weeks

Provinces		Models	With unadjusted ${R}_{t}$			With adjusted ${R}_{t}$		
			${R}^{2}$	%△${R}^{2}$	df	${R}^{2}$	%△${R}^{2}$	df
Northern China	Beijing	Model1^a^	0.39	-	719	0.13	-	719
		Model2^b^	0.43	5.00	711	0.25	12.00	711
		Model3^c^	0.52	13.00	677	0.54	41.00	677
	Tianjin	Model1^a^	0.50	-	663	0.33	-	663
		Model2^b^	0.52	2.00	655	0.50	17.00	655
		Model3^c^	0.61	11.00	621	0.71	38.00	621
	Gansu	Model1^a^	0.42	-	488	0.03	-	488
		Model2^b^	0.46	4.00	484	0.37	34.00	484
		Model3^c^	0.56	14.00	450	0.48	45.00	450
	Liaoning	Model1^a^	0.08	-	623	0.06	-	623
		Model2^b^	0.21	13.00	615	0.39	33.00	615
		Model3^c^	0.28	20.00	587	0.45	39.00	587
Southern China	Shanghai	Model1^a^	0.64	-	828	0.40	-	828
		Model2^b^	0.65	1.00	820	0.42	2.00	820
		Model3^c^	0.68	3.00	800	0.45	3.00	800
	Jiangsu	Model1^a^	0.43	-	831	0.04	-	831
		Model2^b^	0.46	3.00	824	0.45	41.00	824
		Model3^c^	0.47	4.00	818	0.43	39.00	818
	Guangdong	Model1^a^	0.20	-	800	0.07	-	800
		Model2^b^	0.25	5.00	792	0.26	19.00	792
		Model3^c^	0.39	19.00	768	0.49	42.00	768
	Hunan	Model1^a^	0.28	-	814	0.35	-	814
		Model2^b^	0.30	2.00	806	0.56	21.00	806
		Model3^c^	0.38	10.00	778	0.51	16.00	778

${R}^{2}$ and $df$ are measures of R-square and degree of freedom from the regression model respectively

${\%\Delta R}^{2}$ measured the change in the explained variance (i.e., variance explained by either model 2 or model 3) in comparison to the model 1. For model 2, the equation is: $\% {\Delta R}^{2}=|({R}_{model2}^{2}-{R}_{model1}^{2})|\times 100$. For model 3, the equation is: $\% {\Delta R}^{2}=|({R}_{model3}^{2}-{R}_{model1}^{2})|\times 100$

^a^ Model1: factors affecting ${R}_{t}$ (or adjusted ${R}_{t}$) include depletion of susceptibles, and /or inter-epidemic factors

^b^ Model2: model 1 for ${R}_{t}$ plus O_3_

^c^ Model3: model 1 for ${R}_{t}$ plus O_3_ and other drivers

## Discussion

Our study, which used data from 2013 to 2018 across eight provinces, revealed significant variations in influenza epidemics and highlighted a significant association between O_3_ concentrations and influenza transmissibility. In areas with high O_3_ levels, we observed a U-shaped relationship with ${R}_{t}$, while an L-shaped association was noted in regions with lower O_3_. The consistent influence of O_3_ across all provinces underscores its pervasive role in influenza dynamics. Our multivariate regression emphasized the important effect of O_3_ on ${R}_{t}$, even when accounting for other factors. These findings will enhance our understanding of the objective relationships between ambient pollutants, especially O_3_, and the prevention and control of influenza epidemic.

Our results support the evidence of earlier work [11] on ambient O_3_ and influenza transmissibility showing a significant negative association. The combined data analysis for the eight provinces showed a U-shaped association between O_3_ and ${R}_{t}$; this U-shaped association was observed for Tianjin, Beijing, Shanghai, and Jiangsu, while in Gansu, Liaoning, Guangdong, and Hunan, the association was L-shaped. An L-shaped association is consistent with the findings of Ali et al. [11], they studied the association between ${R}_{t}$ and ambient O_3_ across all the types/subtypes of influenza. To our knowledge, this is the first study to report a U-shaped association between O_3_ and ${R}_{t}$_._

Differences in the shape of the association between O_3_ and ${R}_{t}$ in may be related to the variances in ambient O_3_ concentrations. At low concentrations, O_3_ and ${R}_{t}$ are more likely to show an L-shaped association. For example, the maximum O_3_ concentration in Hong Kong did not exceed 140 µg/m^3^ in Ali et al.'s research [11] and the maximum O_3_ concentration did not exceed 180 µg/m^3^ in Gansu, Liaoning, Guangdong, and Hunan. In contrast, the U-shaped association between O_3_ and *R*_*t*_ may become more visible at high concentrations of O_3_ exposure. The maximum O_3_ concentrations exceeded 180 µg/m^3^ in Shanghai and Jiangsu, and those in Beijing and Tianjin exceeded 200 µg/m^3^. These four provinces showed a U-shaped association. Our findings are consistent with the conclusion of Wang et al. that exposure to both low concentration and extremely high concentration of ambient O_3_ increased the risk of influenza [27]. These results consistently reflect that there may be a U-shaped concentration-reaction correlation between O_3_ and influenza/ influenza transmissibility, and suggest that attention should be given to the causes of high ambient O_3_ levels, as appropriate measures to reduce these may be beneficial for reducing influenza transmission.

Similar to previous research [11], our multivariate regression analysis showed that a large proportion of ${R}_{t}$ variance was explained by the intrinsic factors in model 1, and ambient O_3_ contributed a further 1%–13% of the total variance. Two main reasons may be responsible for the U-shaped association between O_3_ and influenza transmissibility. First, reductions in influenza transmissibility may be related to the virucidal activity of O_3_ and its effect on host defense. In vitro studies have reported that O_3_ can inactivate the influenza virus within hours [28], and animal toxicological studies demonstrate that inflammation, injury, and oxidative stress are reduced following exposure to O_3_ at concentrations as low as 300 ppb (642$ug/{m}^{3}$)for up to 72 h [29]. In mice, continuous exposure to 0.5 ppm (1072$ug/{m}^{3}$)O_3_ could reduce the lung injury induced by influenza via activate the immune suppression mechanism [30]. Inhalation of ambient O_3_ has also been shown to enhance type-2 immune responses that promote allergy- and asthma-related responses in healthy human subjects and susceptible populations [31]. Second, higher influenza transmissibility may be associated with a positive relationship between short-term, high-concentration O_3_ exposure and respiratory infection. For example, previous studies of animals provide evidence for increased susceptibility to pneumonia infection after exposure to high concentrations (2 ppm: 4885 $ug/{m}^{3}$) of O_3_ [32]; and animal studies also report increased injury markers and inflammatory responses following O_3_ exposure at concentrations of 1 ppm (2142$ug/{m}^{3}$)or more [33, 34].

There are some potential limitations in this study. First, the seasonal influenza data were collected from surveillance sentinel hospitals, and values varied between years, which could have negatively affected the results. Second, observations from other parts of the world would help evaluate the studied associations in other climatic settings and populations. Third, we interpolated daily incidence rates from the weekly data, which may artificially reduce variability and lead to underestimated effects. Thus, where available, using daily positive ILI rate data would likely prove advantageous.

## Conclusions

From 2013–2018, 54 influenza epidemics were studied across eight provinces. A significant correlation was found between O_3_ concentrations and influenza transmissibility. High O_3_ regions showed a U-shaped relationship with transmissibility, while low O_3_ areas had an L-shaped association. This U-shaped finding is novel, emphasizing O_3_'s role in influenza dynamics. In various climatic conditions, this study provides supplemental evidence regarding the impact of O_3_ on influenza, enriching research on environmental factors driving influenza variations. These findings could be instrumental for public health strategies, suggesting the need to surveillance and manage ambient O_3_ levels to mitigate influenza spread.

### Supplementary Information

**Additional file 1. **

## Data Availability

Due to the potentially sensitive information included, the original dataset is not public and is available from the corresponding author upon reasonable request. The authors declare no competing interests.

## Abbreviations

O_3_: Ozone
ILI: Influenza-like illness (ILI)
$R_t$: Instantaneous reproduction number
