# Supplementary material for: Association between ozone and influenza transmissibility in China
Source: BMC Infect Dis. 2023 Nov 6;23:763. doi: 10.1186/s12879-023-08769-w (PMC10626750; doi:10.1186/s12879-023-08769-w)
Supplement: Supplementary file 1 — Additional file 1. [file 12879_2023_8769_MOESM1_ESM.docx]

**Supplementary Information –Association between Ozone and Influenza Transmissibility in China**

Jiao Yang^1,2,3,#^, Ting Zhang^1,2,3,#^, Liuyang Yang^4,1,2,3,#^, Xuan Han^1,2,3^, Xingxing Zhang^1,2,3^, Qing Wang^1,2,3^, Luzhao Feng ^1,2,3, *^, Weizhong Yang^1,2,3, *^

^1^ School of Population Medicine and Public Health, Chinese Academy of Medical Sciences & Peking Union Medical College, Beijing, China

^2^ State Key Laboratory of Respiratory Health and Multimorbidity, Beijing, China

^3^ Key Laboratory of Pathogen Infection Prevention and Control (Peking Union Medical College), Ministry of Education

^4^ Department of management science and information system, Faculty of Management and Economics, Kunming university of science and technology, Kunming, Yunnan, China

^*^ Corresponding author at: School of Population Medicine and Public Health, Chinese Academy of Medical Sciences & Peking Union Medical College, Beijing, China.

*E-mail address:* yangweizhong@cams.cn (W.Yang), fengluzhao@cams.cn (L.Feng).

^#^ These authors contributed equally to the study.

**CONTENTS**

**1. Estimation of Transmissibility**

**2. Construction of Multivariable Regression Models**

**3. Derivation of adjusted**$\boldsymbol{R}_{\boldsymbol{t}}$

**4. Supplementary tables or figures**

*4.1Table S1.*

*4.2Table S2.*

*4.3Table S3.*

*4.4 Fig.S1*

**5. References**

1. Estimation of Transmissibility

Based on the branching process model proposed by Cori et al. within the Bayesian framework[1], the weekly instantaneous reproduction number $R_{t}$ was estimated.This is an extension of Fraser method[2]. Fraser suggested that the renewal estimation equation for the $R_{t}$ of an epidemic could be as

$$R_{t}=\frac{I_{t}}{\sum_{s=0}^{m} w_{s}I_{t-s}} (1)$$

where $I_{t}$ refers to the number of reported cases (specifically, the incidence rate multiplied by a constant 10,000) between time $t$and time $t+1$.The term $w_{s}$ denotes the generation time distribution, satisfying $\sum_{s=0}^{m} w_{s}=1$. The expected incidence at time $t$ follows a Poisson distribution with a mean ($R_{t}\sum_{s=0}^{m} w_{s}I_{t-s}$). Transmissibility is assumed constant over the interval [$t-\tau, t$] and is quantified by the $R_{[t-\tau,t]}$.The likelihood of $I_{t-\tau}, \ldots\ldots\ldots,I_{t}$ given the reproduction number $R_{\left[ t-\tau,t \right]}$ and $I_{0}, \ldots\ldots\ldots,I_{t-\tau-1}$ is as follows:

$${P(I}_{t-\tau}, \ldots\ldots\ldots,I_{t}\left| I_{0}, \ldots\ldots\ldots,I_{t-\tau-1}, w,R_{\left[ t-\tau,t \right]}) \right.=\prod_{s=t-\tau}^{t} \frac{{{e^{-R_{\left[ t-\tau,t \right]}\Lambda_{s}} (R}_{\left[ t-\tau,t \right]}\Lambda_{s})}^{I_{s}}}{I_{s}!} (2)$$

where, $\Lambda_{s}=\sum_{s=0}^{m} w_{s}I_{t-s}$. A gamma distribution, characterized by a mean of 2.6 days and a standard deviation of 1.5 days, was assumed to represent the generation time. This assumption remains constant throughout an epidemic. A Bayesian framework was developed using a Gamma-distributed prior with parameters (a, b) for$R_{\left[ t-\tau,t \right]}$. Consequently, the posterior joint distribution of$R_{\left[ t-\tau,t \right]}$ can be derived as proportional to

$${{R_{\left[ t-\tau,t \right]}(posterior)=R}_{\left[ t-\tau,t \right]}}^{a+\sum_{s=t-\tau}^{t} I_{s}-1} e^{-R_{\left[ t-\tau,t \right]}(\sum_{s=t-\tau}^{t} \Lambda_{s}+\frac{1}{b})} \prod_{s=t-\tau}^{t} \frac{{\Lambda_{s}}^{I_{s}}}{I_{s}!} (3)$$

Equation (3) indicates that the posterior distribution of $R_{\left[ t-\tau,t \right]}$is a gamma distribution with the parameters$(a+\sum_{s=t-\tau}^{t} I_{s},{(\sum_{s=t-\tau}^{t} \Lambda_{s}+\frac{1}{b})}^{-1})$.

**2. Construction of Multivariable Regression Models**

As described by Ali et al[3], the transmissibility of influenza is generally influenced by the depletion of susceptibles and inter-epidemic effects as intrinsic drivers, along with the potential effects of the extrinsic drivers (e.g. O_3_ and meteorological drivers). Therefore, general multivariable nonlinear regression models were developed to examine the underlying association between influenza transmissibility and different various drivers.

Drawing to the previous research[3, 4], we found that the exponential, represented by $R_{t}=e^{\varphi(f_{k})}$, where, $\varphi\left( f_{k} \right)=\beta_{2}{f_{k}}^{2}+\beta_{1}f_{k}+\beta_{0}$, to be a superior fit for capturing associations across all drivers of influenza transmissibility (Table S2). Here, $\beta_{i}$ denotes the corresponding coefficients, $f_{k}$ indicates the $k$-th drivers.

Accordingly, the instantaneous reproduction number $R_{ij}$ can be expressed as,

$$R_{ij}=R_{0}S_{ij}\prod_{k} e^{\varphi(a_{ijk} )} (2.1)$$

Here, $R_{ij}$ denotes time-varying instantaneous reproduction number on the $i-$th day of the$j-th epidemic$; $S_{ij}$ represents the fraction of susceptibles at the commencement of the $i$th day/weeks of $j$th epidemic. While $R_{0}$ signifies the basic reproduction number. The impact of the k-th driving variables is symbolized by$a_{ijk}$. The values of $S_{ij}$ is derived subtracting the cumulative number of infections from the fraction of the population initially susceptible at the onset of the influenza season ($S_{0j}$).

Let $h_{ij}$ represent the observed cumulative incidence up to the (i-1)-th day of the j-th epidemic. This can be expressed as:

$h_{ij}=\sum_{x}^{i-1} I_{xj}$, where $I_{xj}$ denotes the incidence on day $x$ of epidemic $j$.

Consequently, the relationship for$S_{ij} is$:

$S_{ij}=S_{0j}-{h_{ij}= S}_{0j}\left( 1+z_{j} h_{ij} \right)$

where $z_{j} is defined as:$

$$z_{j} ={-c_{i}/S}_{0j}$$

and for the given context, the constant$c_{i}$ equals 1

Using the Taylor series expansion, we can represent $S_{ij} as:$

$$S_{ij}\approx S_{0j}e^{z_{j} h_{ij}}$$

From this, we derive:

$$R_{ij}=R_{0}S_{0j}e^{z_{j} h_{ij}} \prod_{k} e^{\varphi(a_{ijk} )} （2.2）$$

When we take the logarithm and approximate the logarithm of the effective reproduction number, we get:

$$ln\left( R_{ij} \right)=\ln\left( R_{0}S_{0j} \right)+z_{j} h_{ij}+\sum_{k} \varphi(a_{ijk}){+ \epsilon}_{ij}$$

Here, we treat the parameters $\ln\left( R_{0}S_{0j} \right)$ and $z_{j}$ as nuisance parameters, indicative of inter-seasonal factors and not immediately pertinent. The error term is given by $\epsilon_{ij}\sim N(0,\sigma^{2})$.

By suitably substituting the terms, the regression equation is formulated as:

$$ln\left( R_{ij} \right)=\alpha_{j}s_{j}+\eta_{j}h_{ij} +\sum_{k} \varphi(a_{ijk})+\epsilon_{ij} (2.3)$$

In this equation, $s_{j}$ denotes the epidemic$j$, a categorical variable with regression coefficient $\alpha_{j}. Meanwhile,$ $\eta_{j}$ represents the regression coefficients that describe the rate at which susceptibles are reduced in the population.

A basic model is defined based on intrinsic factors only. i.e. $ln\left( R_{ij} \right)=\alpha_{j}s_{j}+\eta_{j}h_{ij}+\epsilon_{ij}$. The $\Delta R^{2}$ measures which compare the R-square values of these models, highlight the variance in transmissibility explained by respective drivers.

**3. Derivation of adjusted**$\boldsymbol{R}_{\boldsymbol{t}}$

The adjusted $R_{t}$ by removing the impact of the depletion of susceptibles from$R_{t}$ using the approach outlined by Ali et al[3]. It's important to note that $R_{t}$ is influenced by both the basic reproduction number ($R_{0}$) and the depletion of susceptibles ($S_{t}$), with its trend declining as the epidemic progresses. We initiated our process by fitting a model: $ln\left( R_{t} \right)=\ln\left( R_{0}S_{0} \right)+\xi h_{t}$ to the estimated$R_{t}$ for each epidemic individually. Here,$R_{0}$ denotes the basic reproduction number. $S_{0j}$ represents the initial susceptible proportion for epidemic $j$. $h_{ij}$ signifies the observed cumulative incidence up to $(i-1)$th day of $j$th epidemic. Furthermore, $\xi is \mathrm{givent} by{-c_{i}/S}_{0j}$, with a constant$c_{i}$, and in this context, $c_{i}=1$. The adjusted $R_{t}$ was then defined as the residual of the fit. Hence, our measures of adjusted $R_{t}$ is devoid of the effects of depletion of susceptibles and assumed to be driven by both inter-epidemic effects and the extrinsic drivers.

Table S1 $\Delta AIC$ values for two different forms of univariate regression models to identify the best model representing the association between instantaneous reproduction number ($R_{t}$) for drivers of influenza.

|  | | Forms of Association | Relative humidity | Absolute humidity | O_3_ |  |
| --- | --- | --- | --- | --- | --- | --- |
|  |  |  |  |  |  |  |
| Northern China | Beijing | **Exponential** | 0 | 0 | 0 |  |
|  |  | **Power** | 3.50 | 14.82 | 30.60 |  |
|  | Tianjin | **Exponential** | 0 | 0 | 0 |  |
|  |  | **Power** | 1.52 | 14.45 | 24.74 |  |
|  | Liaoning | **Exponential** | 0 | 0 | 0 |  |
|  |  | **Power** | 6.48 | 8.24 | 6.91 |  |
|  | Gansu | **Exponential** | 0 | 0 | 0 |  |
|  |  | **Power** | 8.56 | 10.61 | 18.56 |  |
| Southern China | Shanghai | **Exponential** | 0 | 0 | 0 |  |
|  |  | **Power** | 1.57 | 29.63 | 47.79 |  |
|  | Jiangsu | **Exponential** | 0 | 0 | 0 |  |
|  |  | **Power** | 2.85 | 52.40 | 24.75 |  |
|  | Guangdong | **Exponential** | 0 | 0 | 0 |  |
|  |  | **Power** | 26.26 | 6.98 | 0.06 |  |
|  | Hunan | **Exponential** | 0 | 0 | 0 |  |
|  |  | **Power** | 2.96 | 0.25 | 0.81 |  |

Note：${\Delta AIC}_{i}=\mathrm{AIC}_{i}-\mathrm{AIC}_{min}$, $i=$exponential and power. Temperature may less than 0, and the school holiday is 0 or 1 to represent whether a certain day is a holiday. Therefore, temperature and school holidays do not have power-form results

Table S2 Percentage of the variance of the instantaneous reproduction number ($R_{t}$) explained by O_3_, compared with1000 null/dummy time series, based on a univariate regression model

| Provinces | | With unadjusted $R_{t}$ | | |  | With adjusted $R_{t}$ | | |  |
| --- | --- | --- | --- | --- | --- | --- | --- | --- | --- |
|  |  | $R^{2}$ | Permutation analysis  $R^{2}$ | $P$^†^ |  | $R^{2}$ | Permutation analysis  $R^{2}$ | $P$^†^ | |
| Northern China | Beijing | 14.18 | 0.13 | <0.05 |  | 22.27 | 0.13 | <0.05 | |
|  | Tianjin | 5.09 | 0.14 | <0.05 |  | 22.02 | 0.15 | <0.05 | |
|  | Gansu | 18.07 | 0.20 | <0.05 |  | 33.94 | 0.19 | <0.05 | |
|  | Liaoning | 15.04 | 0.16 | <0.05 |  | 35.26 | 0.16 | <0.05 | |
|  |  |  |  |  |  |  |  |  | |
| Southern China | Shanghai | 6.53 | 0.12 | <0.05 |  | 7.82 | 0.12 | <0.05 | |
|  | Jiangsu | 7.99 | 0.10 | <0.05 |  | 2.46 | 0.11 | <0.05 | |
|  | Guangdong | 2.47 | 0.13 | <0.05 |  | 3.85 | 0.11 | <0.05 | |
|  | Hunan | 1.32 | 0.12 | <0.05 |  | 1.91 | 0.12 | <0.05 | |

^†^ indicating a significant difference between $R^{2}$and Permutation analysis $R^{2}$.

Table S3 Percentage of the variance in the instantaneous reproduction number ($R_{t}$) explained by the drivers, across respective provinces from 2013 to 2018. The results are based on the best lag regression models (with the lag lying highest $R^{2}$-value) using lags ranging from 0 to 2 weeks

| Provinces | | Models | With unadjusted $R_{t}$ | | |  | With adjusted $R_{t}$ | | | |
| --- | --- | --- | --- | --- | --- | --- | --- | --- | --- | --- |
|  |  |  | $R^{2}$ | %△$R^{2}$ | df |  | $R^{2}$ | %△$R^{2}$ | %△$R^{2}$ |  |
| Northern China | Beijing | Model1^†^ | 0.36 | - | 719.00 |  | 0.11 | - | 719 |  |
|  |  | Model2^‡^ | 0.37 | 1 | 711.00 |  | 0.35 | 24 | 711 |  |
|  |  | Model3^*^ | 0.41 | 5 | 677.00 |  | 0.41 | 29 | 677 |  |
|  | Tianjin | Model1^†^ | 0.37 | - | 663.00 |  | 0.09 | - | 663 |  |
|  |  | Model2^‡^ | 0.35 | 2 | 655.00 |  | 0.43 | 34 | 655 |  |
|  |  | Model3^*^ | 0.33 | 4 | 621.00 |  | 0.46 | 37 | 621 |  |
|  | Gansu | Model1^†^ | 0.35 | - | 488.00 |  | 0.04 | - | 488 |  |
|  |  | Model2^‡^ | 0.37 | 2 | 484.00 |  | 0.42 | 38 | 484 |  |
|  |  | Model3^*^ | 0.39 | 4 | 450.00 |  | 0.51 | 47 | 450 |  |
|  | Liaoning | Model1^†^ | 0.23 | - | 623.00 |  | 0.02 | - | 623 |  |
|  |  | Model2^‡^ | 0.24 | 1 | 615.00 |  | 0.72 | 70 | 615 |  |
|  |  | Model3^*^ | 0.25 | 2 | 587.00 |  | 0.63 | 61 | 587 |  |
| Southern China |  |  |  |  |  |  |  |  |  |  |
|  | Shanghai | Model1^†^ | 0.35 | - | 828.00 |  | 0.09 | - | 828 |  |
|  |  | Model2^‡^ | 0.35 | 0 | 820.00 |  | 0.37 | 26 | 820 |  |
|  |  | Model3^*^ | 0.36 | 1 | 800.00 |  | 0.39 | 30 | 800 |  |
|  | Jiangsu | Model1^†^ | 0.39 | - | 831.00 |  | 0.03 | - | 831 |  |
|  |  | Model2^‡^ | 0.43 | 4 | 824.00 |  | 0.47 | 44 | 824 |  |
|  |  | Model3^*^ | 0.44 | 5 | 818.00 |  | 0.43 | 40 | 818 |  |
|  | Guangdong | Model1^†^ | 0.27 | - | 800.00 |  | 0.05 | - | 800 |  |
|  |  | Model2^‡^ | 0.29 | 2 | 792.00 |  | 0.27 | 22 | 792 |  |
|  |  | Model3^*^ | 0.29 | 2 | 768.00 |  | 0.52 | 47 | 768 |  |
|  | Hunan | Model1^†^ | 0.19 | - | 814.00 |  | 0.33 | - | 814 |  |
|  |  | Model2^‡^ | 0.18 | 1 | 806.00 |  | 0.52 | 19 | 806 |  |
|  |  | Model3^*^ | 0.18 | 1 | 778.00 |  | 0.51 | 18 | 778 |  |

^†^ Model1: factors affecting $R_{t}$ (or adjusted$R_{t}$) include depletion of susceptibles, and /or inter-epidemic factors.

^‡^ Model2: model 1 for $R_{t}$plus O_3_.

^*^ Model3: model 1 for $R_{t}$plus O_3_ and other drivers.

$R^{2}$ and $df$ are measures of R-square and degree of freedom from the regression model respectively.

${\%\Delta R}^{2}$ measured the change in the explained variance (i.e., variance explained by either model 2 or model 3) in comparison to the model 1. For model 2, the equation is: $\% {\Delta R}^{2}=|(R_{model2}^{2}-R_{model1}^{2})|\times100$. For model 3, the equation is: $\% {\Delta R}^{2}=|(R_{model3}^{2}-R_{model1}^{2})|\times100$.


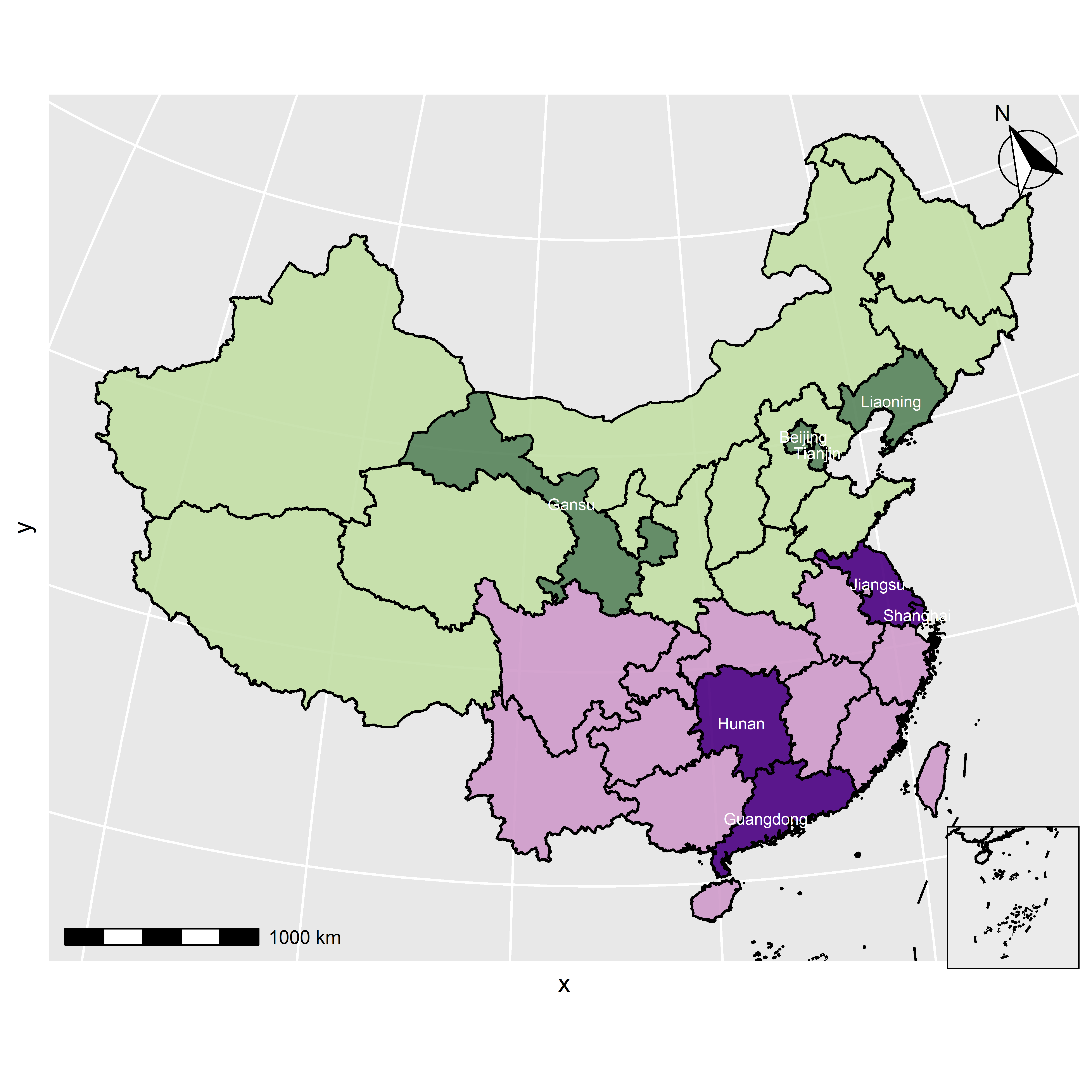


Fig. S1. The map indicating 8 provinces studied in northern China (dark green) and southern China (dark purple).

**5. References**

1. Cori A, Ferguson NM, Fraser C, Cauchemez S: **A new framework and software to estimate time-varying reproduction numbers during epidemics**. *Am J Epidemiol* 2013, **178**(9):1505-1512.

2. Fraser C: **Estimating individual and household reproduction numbers in an emerging epidemic**. *PLoS One* 2007, **2**(8):e758.

3. Ali ST, Cowling BJ, Wong JY, Chen D, Shan S, Lau EHY, He D, Tian L, Li Z, Wu P: **Influenza seasonality and its environmental driving factors in mainland China and Hong Kong**. *Sci Total Environ* 2022, **818**:151724.

4. te Beest DE, van Boven M, Hooiveld M, van den Dool C, Wallinga J: **Driving factors of influenza transmission in the Netherlands**. *Am J Epidemiol* 2013, **178**(9):1469-1477.
